# Enabled or Disabled: Is the Environment Right for Using Biodiversity to Improve Nutrition?

**DOI:** 10.3389/fnut.2016.00014

**Published:** 2016-06-06

**Authors:** Danny Hunter, Isa Özkan, Daniela Moura de Oliveira Beltrame, Wellakke Lokuge Gamini Samarasinghe, Victor Wafula Wasike, U. Ruth Charrondière, Teresa Borelli, Jessica Sokolow

**Affiliations:** ^1^Bioversity International, Rome, Italy; ^2^General Directorate of Agricultural Research and Policy, Ministry of Food, Agriculture and Livestock, Ankara, Turkey; ^3^Biodiversity for Food and Nutrition Project, Brasilia, Brazil; ^4^Plant Genetic Resources Centre, Peradeniya, Sri Lanka; ^5^Kenya Agricultural and Livestock Research Organization, Nairobi, Kenya; ^6^Food and Agriculture Organization of the United Nations, Rome, Italy

**Keywords:** biodiversity, environment, nutrition, agriculture, Convention on Biological Diversity, Biodiversity for Food and Nutrition Project (BFN)

## Abstract

How can we ensure that 9 billion people will have access to a nutritious and healthy diet that is produced in a sustainable manner by 2050? Despite major advances, our global food system still fails to feed a significant part of humanity adequately. Diversifying food systems and diets to include nutrient-rich species can help reduce malnutrition, while contributing other multiple benefits including healthy ecosystems. While research continues to demonstrate the value of incorporating biodiversity into food systems and diets, perverse subsidies, and barriers often prevent this. Countries like Brazil have shown that, by strategic actions and interventions, it is indeed possible to create better contexts to mainstream biodiversity for improved nutrition into government programs and public policies. Despite some progress, there are few global and national policy mechanisms or processes that effectively join biodiversity with agriculture and nutrition efforts. This perspective paper discusses the benefits of biodiversity for nutrition and explores what an enabling environment for biodiversity to improve nutrition might look like, including examples of steps and actions from a multi-country project that other countries might replicate. Finally, we suggest what it might take to create enabling environments to mainstream biodiversity into global initiatives and national programs and policies on food and nutrition security. With demand for new thinking about how we improve agriculture for nutrition and growing international recognition of the role biodiversity, the 2030 Agenda for Sustainable Development presents an opportunity to move beyond business-as-usual to more holistic approaches to food and nutrition security.

## Introduction

Despite considerable strides in feeding the world’s population, food systems still fall short of doing so in a healthy or environmentally friendly manner. Malnutrition remains a grand global challenge. Globally, approximately 795 million people are undernourished (1) and many people suffer from one or more micronutrient deficiencies, such as iron deficiency, which the World Health Organization (WHO) estimates affects 30% of the world’s population.^1^ Furthermore, 2.1 billion people are estimated to be overweight or obese (2). The global food system is also a major contributor to climate change and environmental degradation including biodiversity loss (3–5). All this is aggravated by the fact that agriculture, food systems and diets are becoming more uniform and simplified, and agriculture remains focused on increasing the production of a narrow number of staple crops and animal species (6). Much of our food biodiversity has been neglected or lost, yet it has huge potential to provide the natural richness of nutrients humans require (7).

These challenges have prompted calls for new thinking and approaches to better integrate biodiversity for improved nutrition including a resurgence of interest among some donors, policy makers, researchers, practitioners, and consumers – accompanied by numerous high-level intergovernmental meetings – in finding ways to reshape food systems that improve nutrition outcomes. Agencies, including the Food and Agriculture Organization of the United Nations (FAO), the Convention on Biological Diversity (CBD), the WHO, and Bioversity International, recognize the important role of biodiversity in this momentum to reshape food systems (8). At the same time, there has been significant global focus on improving agriculture for enhanced nutrition outcomes, often referred to as “nutrition-sensitive agriculture,” which has involved numerous partners under the leadership of the United Nations Standing Committee on Nutrition (UNSCN) as well as within the CGIAR Consortium, in particular, through its Research Program on Agriculture for Nutrition and Health (A4NH) (9).

Despite these developments, there is no global and few national political mechanisms or processes that effectively bring together the environment, agriculture and nutrition sectors (with the environment sector responsible for biodiversity), and which might facilitate more coherent horizontal coordination between policy and programing. The International Assessment of Agricultural Knowledge, Science, and Technology for Development (IAASTD) highlights the need for better integrative and holistic approaches (10), as does more recently, the International Panel of Experts on Sustainable Food Systems (11). Notwithstanding the rhetoric around new thinking and approaches, there still appears limited appetite for promoting biodiversity for improved nutrition, and we are certainly a long way from having an ideal enabling environment that might make that appetite a little bit healthier. Why is this so, despite what we know about the multiple benefits of biodiversity including improving diets and nutrition?

## What are the Benefits of Biodiversity for Nutrition?

Biodiversity and health are tightly bound (12–14) with biodiversity important for food security and nutrition including the provision of macronutrients, micronutrients, and bioactive non-nutrients for healthy diets (7, 15, 16). Taking a whole diet approach, a holistic method to identify dietary patterns and nutritional gaps enables the use of different combinations of diverse foods, and their many interactions, to improve dietary quality and meet nutritional needs.

The dependence and impact of human nutrition on biodiversity is increasingly acknowledged by the health, agriculture, and environment sectors and includes a range of research, program, policy, and advocacy efforts. Despite this, agricultural production continues to focus on a few staple crops, and its impact is measured in food quantity or dietary energy supply, which does not automatically ensure appropriate nutritional quality (8). Such metrics are inadequate in measuring the production of healthy foods that contribute to sustainable diets and meet individual nutritional needs (17).

Despite growing awareness of the benefits of biodiversity for diverse diets and the nature of diet-related nutrition and health problems, many barriers and perverse subsidies hinder mainstreaming biodiversity considerations (11, 18). We live in a time when national and global food supplies are becoming more homogeneous in composition, largely dependent on a few global crops (6). Furthermore, the international nutrition and health community tends to focus on technological fixes such as supplements, fortification, and biofortification as a solution to nutritional problems, where biodiverse food-based approaches could be a more sustainable solution or part of it. Disentangling these barriers is beyond the scope of this perspective paper, yet it might be instructive to highlight just one of the main barriers. Malnutrition is a multisectoral challenge; there is no single cause, no single solution. Yet, some sectors, which can and should contribute to finding sustainable solutions, continue to function in silos. The disconnect between the relevant sectors – agriculture, environment, health, and nutrition – has led to much policy and programing incoherence and what could be even considered a disabling environment for the promotion of biodiversity for improved nutrition (19).

## What Does an Enabling Environment for Biodiversity to Improve Nutrition Look Like?

*Leveraging Agriculture for Nutrition in South Asia* (LANSA), an international research partnership, seeks to explore how agriculture and food systems can be better designed to enhance nutrition. It considers environments in which the basic social, economic, and political conditions are broadly favorable to nutrition as “enabling environments.” In contrast, where such environments are not conducive, they are considered as neutral or even disabling for nutrition.^2^ As a starting point, LANSA and other partners undertook a survey of enabling environments (20) and what this meant outside of agriculture and formulated a framework of three domains on which to focus:

- *Evidence base* – including the framing of evidence and communication of different forms of evidence;
- *Politics and governance* – especially cross-sectoral coordination (including both horizontal and vertical coordination) bringing together the agriculture and health sectors; and
- *Capacity building and financing* – exploring the potential of agricultural services to take on board nutrition and determine if nutrition-sensitive agriculture costs more than conventional agriculture.

Gillespie et al. (20) use the definition of an enabling environment “*as political and policy processes that build and sustain momentum for the effective implementation of actions that reduce undernutrition*.”

**Figure 1:**
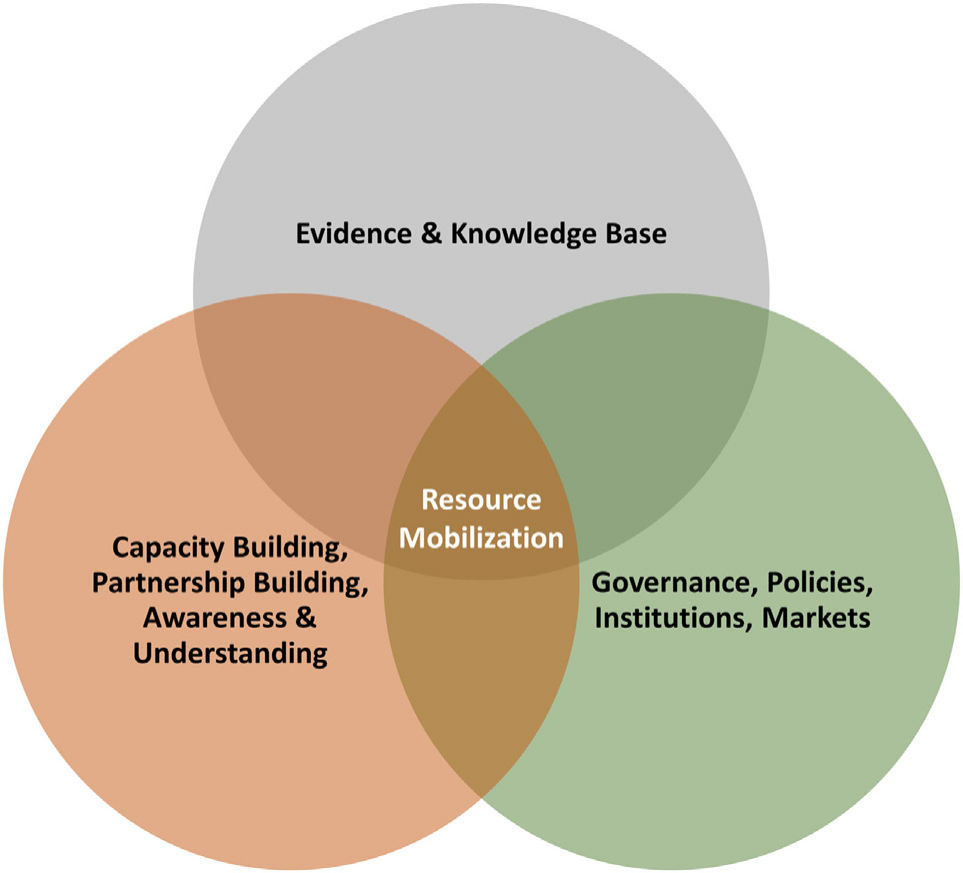
**The main domains of an enabling environment for BFN**.

Mirroring the focus of the LANSA experience, the *Mainstreaming Biodiversity Conservation and Sustainable Use for Improved Nutrition and Well-Being* (Biodiversity for Food and Nutrition^3^ – or BFN for short) project, a Global Environment Facility (GEF) funded project working through national environment and agriculture sectors, has been developing a program of work to establish enabling environments for the purpose of mainstreaming biodiversity for food and nutrition, and which identifies three components that have much in common with the three LANSA domains (see Figure 1).

Do such general frameworks, and their specific elements, provide enough guidance to integrate biodiversity into relevant global and national food security and nutrition policies and programs? If so, what is the way forward that might bring relevant sectors together to create better enabling environments, and lead to improved policy, program, and resource mobilization coherence? Gillespie et al. (20) point out that each context needs its own enabling narrative or framing. What is the enabling framing and definition for the mainstreaming of biodiversity for improving nutrition?

## How Can We Create an Enabling Environment for Biodiversity to Improve Nutrition?

What follows in this section is a brief survey of some recent activities and initiatives by BFN project countries – Brazil, Kenya, Sri Lanka, and Turkey – to facilitate processes and actions to create momentum, which promotes the use of biodiversity for improving nutrition.

### Improving the Knowledge and Evidence Base

Scientific evidence demonstrates that biodiversity is critical to human health and well-being (14, 21), including for improved dietary diversity and nutrition (16). The literature reports significant intraspecific differences in the nutrient content of most plant-source foods and other relevant edible biodiversity (22, 23), which are often nutritionally significant. However, significant knowledge and evidence gaps need to be addressed if we seriously wish to transform the enabling environment for biodiversity to improve nutrition (24, 25). Among these are the need for more food composition data (as data currently exist for only a fraction of the world’s edible biodiversity), dietary intake data and better research to understand the complex pathways that link biodiversity to nutrition and health (8).

While it is understood that these gaps will take time and money to address, progress can and is being made. As part of the BFN Project, an alliance of universities and government agencies are undertaking comprehensive nutritional analysis of over 150 priority species and compiling national information systems to make available and communicate this knowledge to relevant sectors and decision makers. While also contributing to the global FAO/INFOODS Food Composition Database for Biodiversity (International Network of Food Data Systems of the Food and Agricultural Organization) (22). For example, Brazil is in the process of establishing the nutritional composition data of over 70 native species, which have been prioritized by the national *Plants for the Future* initiative. This information will be made available through the national Information System on Brazilian Biodiversity (SiBBr) and will strengthen the inclusion of nutritious species in public policies and programs focused on food security and nutrition including public procurement and school feeding.

### Improving Policies and Governance

The 2014 State of Food Insecurity in the World report highlights the advances countries such as Brazil have made in reducing hunger and strengthening food security. The Zero Hunger Strategy and associated public policies are singled out as being at the forefront of Brazil’s fight against hunger and food insecurity while contributing to strengthening family farming, inclusive rural development, and improving accessibility to food through various social protection measures. Brazil, through the BFN initiative has made considerable strides in promoting biodiversity for improved nutrition by taking advantage of existing horizontal cross-sectoral governance mechanisms created or strengthened under the Zero Hunger Strategy framework and targeting relevant public policies (26). In Brazil, BFN prioritized four existing public policies – the Food Acquisition Program (PAA), the National School Meals Program (PNAE), the National Food and Nutrition Policy (PNAN) and the Minimum Price Guarantee Policy for Biodiversity Products (PGPM-Bio) – which benefit from data on the nutritional value of native biodiversity and provide entry points for improving nutrition.

Countries can also take advantage of other national multisectoral coordinating platforms already in place. *The Scaling Up Nutrition* (SUN) movement that started in 2010 seeks to promote horizontal coherence through the establishment of national multisectoral platforms to facilitate coordinated and integrated action (20), but seldom considers the importance of biodiversity for improving nutrition. Coordination between biodiversity initiatives, such as BFN, and multisector initiatives, such as SUN, can help to bridge these gaps and foster an enabling environment. In 2014, the BFN initiative in Kenya was invited to join the Nutrition Interagency Coordinating Committee (NICC), a multi-stakeholder platform chaired by the Ministry of Health and SUN Focal Point. According to BFN partners, participating in committees, such as the NICC and SUN, provides a major opportunity to embed biodiversity in already established national coordination mechanisms and to link biodiversity and agriculture to nutrition as an option for addressing nutritional issues.

Ensuring that horizontal coordination leads to action and results at the local level (vertical coherence) is another important consideration. This is especially important given that many countries in Asia and sub-Saharan Africa are moving to decentralized systems of governance (20). This requires that local governments, districts, counties, and municipalities have the relevant policies, programs, capacities and resources to deliver on any plans to promote biodiversity for improved nutrition. Kenya is one example where such political, administrative, and financial decentralization is taking place, where opportunities to mainstream biodiversity are currently being explored at the county level. BFN is presently assisting the Busia County government to develop a policy on biodiversity conservation and utilization with links to agriculture and biodiversity, a first for any of Kenya’s 47 counties.

### Improving Capacity, Partnerships, and Awareness

Realizing effective enabling environments requires significant attention to novel ways to build capacity, partnerships and alliances, and improving awareness and understanding among many actors. This includes working with universities to encourage the necessary interdisciplinary approaches to teaching and research. Partnerships with federal universities in Brazil ensures that nutritional composition methodology developed by FAO/INFOODS is promoted and embedded in teaching and research across a range of regional universities and research institutes. Activities are underway to support this through the development of online modules to strengthen capacity to mainstream biodiversity for improved nutrition. Modules will target professionals responsible for public policy development and those providing technical support to the implementation and execution of government initiatives related to food and nutrition security, at federal, state, and municipal levels. Universities involved in this partnership also host the Collaboration Centers on Food and Nutrition (CECANEs) that provide capacity and technical research support and guidance to those involved in delivering the national school meals program under PNAE, which feeds over 40 million children per day and creates opportunities for the inclusion of biodiversity in school meals (26).

## Mobilizing for Nutrition: Can Biodiversity be Harnessed?

The financial resources required to address malnutrition are considerable (25). Overseas nutrition-related spending (in the amount of aid) only helps meet approximately 1.4% of the total amount required to accelerate progress in reducing undernutrition. This figure does not capture government commitments and is likely not indicative of the full picture of donor funding, given the multisectoral nature of nutrition activities.^4^

Countries that have multisectoral plans in place to address malnutrition rarely include the environment sector among the usual “nutrition-relevant sectors” that are invited to participate, possibly missing opportunities. Yet, biodiversity, as an identified basic determinant of nutrition status, is highlighted as a potentially important sector for nutrition, especially for the poorest, and as a contributor to dietary diversity (25).

How might the environment sector raise the profile of biodiversity as a contributor to improving nutritional outcomes? One approach is through National Biodiversity Strategy and Action Plans (NBSAPs). The majority of countries that have multisectoral nutrition plans or who are members of SUN are also signatories to the CBD and are required to integrate the conservation and sustainable use of biodiversity into relevant national sectoral or cross-sectoral plans, programs, and policies. As already highlighted, Brazil is one country that has attempted to bring about broad horizontal coordination to address nutrition and which has more recently been inclusive of the environment sector. It is also a country that has made significant efforts to align its NBSAP process to highlight the importance and value of biodiversity to address nutrition. NBSAPs are national instruments that signatory countries are required to develop in order to meet their obligations to the CBD and are key for mainstreaming biodiversity into key development policies, plans, and processes of all sectors that have an impact, positive or negative, on biodiversity. This is probably the first time, in the case of Brazil, that a NBSAP has been revised to include such nutrition-related objectives, targets, and indicators and with dedicated resources and budgets to support implementation of actions (26).

## A Final Word: Enabling Environments and the Global Policy Context

International cooperation linking biodiversity, agriculture and nutrition must improve if it is to drive change at the country level. Despite global efforts to better align agriculture and nutrition goals as well as calls from the environment sector for greater engagement with the health sector and a broader range of organizations interested in health and biodiversity (12, 27), disconnects remain. The CGIAR Research Program A4NH, with a flagship area of work on *Integrated Programs and Policies* and a call for work on cross-sectoral policy processes, neglects the inclusion of the biodiversity community among its strategic enablers at the global, regional and national level. Within the United Nations system similar disconnects can be found. While the CBD and WHO engage on biodiversity and health, the WHO and FAO on agriculture and health, and FAO and CBD on agriculture and biodiversity, there is seemingly little effective horizontal cooperation across all relevant sectors on this issue. Presumably leading to less than ideal policy coherence and missed opportunities. For example, the recent Second International Conference on Nutrition (ICN2) missed a timely opportunity to draw world attention to the important role of biodiversity for improving nutrition. One possible solution for greater coherence could be a role for the CBD in the Committee on World Food Security (CFS), the world’s foremost inclusive international and intergovernmental platform on food security and nutrition. This could help realize greater gains as we move into the 2030 Agenda for Sustainable Development.

At present, the importance of biodiversity for healthy agriculture, food systems and diets is not adequately reflected in the sustainable development goals (SDGs). While all of these elements are addressed in SDG2, there currently are no indicators in that goal that might capture these linkages. To do so, indicators must go beyond conventional measures of agricultural production and yield to better measure nutritional quality (17), nutritional diversity of food systems (28), and dietary diversity (29). More effort is needed to better mainstream biodiversity into relevant SDG indicators, so that the multiple ecosystem goods and services it can deliver for human well-being are better tracked.

There has never been a better time to facilitate a more holistic approach to food security and nutrition involving all relevant sectors, to reverse the decades of unsustainable nutrition-related interventions (30), and to better mainstream biodiversity as one of multiple solutions to malnutrition. In addition to growing demand for new thinking on how we improve agriculture for nutrition, the area of biodiversity, environment, and health, including nutrition, is high on the international agenda (13, 14, 31). Better interdisciplinary analysis and cross-sectoral collaboration, with partnership and involvement from local communities, will be essential. It will require transformative political will, leadership and vision. It would be naive not to mention that it will also require major structural transformations to address the distorted economics of our current food system to better capture negative public health and environment externalities and the true cost of our food. While there has been some convergence between relevant sectors to better comprehend the interdependence between human and environmental health, and how biodiversity plays a role in maintaining both, much more is required to better understand and address nutrition and environmental sustainability. There is a long road yet to travel but the 2030 Agenda for Sustainable Development provides an opportunity to move beyond business-as-usual.

## Author Contributions

All authors contributed to the conception of the manuscript and development of ideas and reviewing drafts; DH wrote the initial draft.

## Conflict of Interest Statement

The authors declare that the research was conducted in the absence of any commercial or financial relationship that could be construed as a potential conflict of interest.

## Footnotes

^1^WHO website. Available at: http://www.who.int/nutrition/topics/ida/en/.

^2^LANSA, Enabling Environments for Nutrition page. Available at: http://lansasouthasia.org/content/enabling-environment-nutrition-0.

^3^Biodiversity for Food and Nutrition Project website. Available at: http://www.b4fn.org/.

^4^Secure Nutrition blog. Available at: https://www.securenutritionplatform.org/Blog/Post.aspx?ID=115.
